# Drivers of hypoglycaemia in anorexia nervosa: Clinical severity, BMI, and illness duration

**DOI:** 10.1192/j.eurpsy.2025.10144

**Published:** 2025-12-17

**Authors:** Alfredo Pulini, Odile Viltart, Mathilde Septier, Daphnée Poupon, Marion Deloulay, Clément Vansteene, Laura Di Lodovico, Philip Gorwood, Philibert Duriez

**Affiliations:** 1 Université Paris Cité Faculté de Santé, France; 2 Universite de Lille Faculte des Sciences et Technologies, France; 3 GHU Paris: Groupe Hospitalier Universitaire Paris Psychiatrie and Neurosciences, France; 4 INSERM U1266: Institute of Psychiatry and Neurosciences of Paris, Paris, France

**Keywords:** anorexia nervosa, body mass index, continuous glucose monitoring, glycaemia, illness duration

## Abstract

**Background:**

Anorexia nervosa (AN) often persists for years, resulting in high morbidity and mortality. Hypoglycaemia, typically assessed from a single morning blood sample, is a critical severity indicator. Continuous glucose monitoring (CGM) provides more comprehensive information on glycaemic patterns. This study aimed to characterize glycaemia in patients with AN and identify its potential drivers among metabolic severity (current BMI), clinical severity (Eating Disorder Inventory-2 [EDI-2] score), and illness duration, in a real-world outpatient setting.

**Methods:**

This cross-sectional study included female outpatients with restricting subtype AN. Participants underwent CGM for five days in their usual environment. Collected data comprised age, BMI, illness duration, EDI-2 score, and continuous glycaemic measurements. Glycaemic biomarkers (hypoglycaemic area under the curve [AUC], mean and minimum glycaemia, and coefficient of variation) were computed over 24-hour periods.

**Results:**

Three hundred and four female patients were monitored for a mean of 4.8 days. No significant correlations were observed between glycaemic biomarkers and BMI. Illness duration was significantly associated with mean and minimum glycaemia (*r* = 0.26 and 0.23, respectively, *p* < 0.001) and with hypoglycaemia AUC (*r* = −0.25, *p* < 0.001).

**Conclusions:**

In female patients with restricting subtype AN, illness duration, rather than BMI, appears to significantly influence glycaemic profiles. This may reflect glycaemic adaptations, a hypothesis that warrants further investigation using CGM, a practical tool for exploring metabolic changes and their potential clinical significance in AN.

## Introduction

Eating disorders typically emerge in adolescence, with peak and median ages of onset at 15 and 18 years, respectively [1]. These disorders often persist for several years and are associated with substantial morbidity and mortality [2]. Anorexia nervosa (AN) is an eating disorder characterized by sustained restriction of energy intake, an intense fear of weight gain, body image disturbance, and excessive physical exercise. In the restricting subtype of AN, patients do not engage in binge-eating or purging behaviours. Metabolic alterations may not only result from chronic undernutrition but also play a significant role in the pathophysiology of this severe disorder [3, 4]. Indeed, metabolic adaptation may enable individuals to maintain a state of chronic undernutrition without acute physiological impairment [4]. Notably, genome-wide association studies have demonstrated a significant negative genetic correlation between AN and glycaemic traits [5]. The persistence of such severe caloric restriction remains enigmatic [6], suggesting that adaptive glycaemic mechanisms may contribute to the emergence and/or maintenance of AN. Consequently, glycaemic metabolism could influence both the severity and duration of the illness.

Hypoglycaemia is an important indicator of clinical severity, typically assessed through fasting blood glucose measurements taken in the morning. In severely malnourished patients, hypoglycaemia can be fatal, particularly in adults [7, 8]. Consequently, several guidelines consider severe hypoglycaemia as a criterion for hospitalization [9]. However, the clinical characteristics associated with hypoglycaemia in AN remain poorly understood. Additional glycaemic markers could provide a more comprehensive assessment of clinical severity, including mean 24-hour glycaemia, daily glycaemic variations, or integrated measures such as cumulative glycaemia captured by the area under the curve. These biomarkers can now be readily obtained using continuous glucose monitoring (CGM), which measures interstitial glucose over several hours or days. Interestingly, modern CGM devices allow users to continue their usual daily activities during monitoring.

Including these devices in the assessment and follow-up of patients with AN, therefore, represents an important advance. Some studies have used CGM to link eating behaviours with glycaemic variations over the entire nycthemeral cycle. However, most research to date has focused on diabetes or obesity [10], while studies in eating disorders have primarily concentrated on bulimia nervosa (BN) and binge-eating disorder (BED) [11–14]. Uotani et al. compared AN and BN in a cohort of 18 patients (4 restricting subtype AN, 9 binge-purging subtype AN, and 5 BN), observing that hypoglycaemia occurred both during the day and at night in AN, whereas it was more frequently observed at night in BN [15]. In the largest sample to date, Germain et al. reported that inpatients with AN spent 21% of the time in a hypoglycaemic state [16]. These studies have been limited to small and heterogeneous samples. Further progress is needed in characterizing and understanding the factors that influence glycaemic fluctuations in AN.

In the present study, we aimed to assess different glycaemia indices and identify their main drivers. We hypothesized that (1) metabolic (current body mass index [BMI]) and clinical severity (Eating Disorder Inventory-2 [EDI-2] total score), and (2) illness chronicity (i.e., duration) would account for a significant proportion of the variability in glycaemic indices in female patients with AN analysed in real-world settings.

## Methods

### Participants

**Figure 1.**
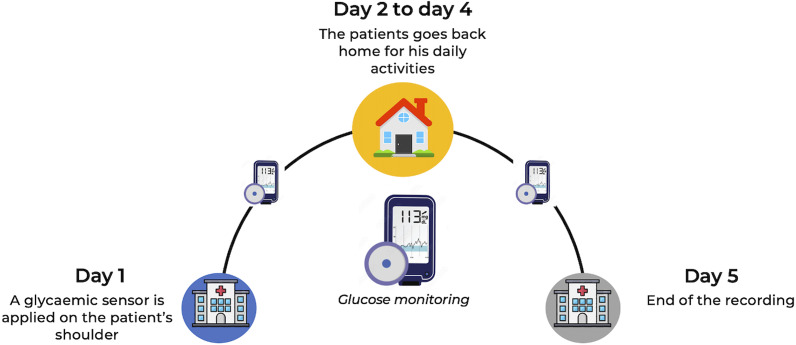
Global clinical evaluation setup for treatment-seeking patients with eating disorders.

This cross-sectional study included adult female outpatients assessed at the *Clinique des Maladies Mentales et de l’Encéphale* (CMME) at GHU Paris Psychiatrie et Neurosciences, France, a university psychiatric centre specialized in the treatment of adult eating disorders. Data collection in this centre has been ongoing since June 2018. Treatment-seeking patients undergo a comprehensive multimodal evaluation, which includes CGM recordings over five days in their usual environment prior to treatment initiation (Figure 1). Written informed consent for the anonymous use of data was obtained from all participants at admission. The study was approved by the GHU Paris Research Ethics Committee on 11 December 2024 (accreditation number 2024-CER-A-023).

BMI was used as an indicator of AN severity. Female patients with restricting subtype AN and a BMI below 18.5 kg/m^2^ were eligible for inclusion. Male patients and those with diabetes were excluded.

### Data collection

Data were extracted from our database using filters to include only female patients with restricting subtype AN, thereby avoiding glycaemic variability related to binge-eating or purging behaviours. The variables selected for this study comprised general characteristics (age, BMI, illness duration), EDI-2 scores, and glycaemic data.

The EDI-2 is a self-report questionnaire containing 91 items rated on six-point Likert scales from 0 to 3, with higher scores indicating more severe disturbances in eating behaviours [17]. Clinical variables were assessed during evaluation by a senior psychiatrist with five years of experience in eating disorders. All patients were diagnosed with AN by clinicians according to DSM-5 criteria [18], following a comprehensive assessment by a psychologist using the Mini-International Neuropsychiatric Interview (MINI) [19] to evaluate all DSM-5 psychiatric disorders, including eating disorders. Glycaemic data were obtained through CGM, as described below.

### Continuous glucose monitoring

Interstitial glucose concentrations were measured using a CGM device (Freestyle Libre Pro®; Abbott Laboratories, Chicago, IL, US). The system comprises sensors that record capillary or interstitial glucose levels continuously over several hours or days, while allowing participants to carry out their usual daily activities. The sensor was applied to the upper arm with a patch in the early afternoon (approximately 13:30) and recorded glucose every 15 minutes, without interfering with daily life. Participants were blinded to their glycaemia data, which were retrieved solely by the nurse at the end of the five-day monitoring period (Figure 1).

### Glycaemic biomarkers

Daily glucose variation was analysed over 24-hour periods, defined from midnight to 23:59. With glucose recorded every 15 minutes, 96 data points could be obtained per day. Four biomarkers were computed: hypoglycaemic area under the curve (AUC), mean glycaemia, minimum glycaemia, and coefficient of variation.

The coefficient of variation, calculated as the standard deviation divided by the mean, provides an estimate of glycaemic variability. Because glucose was recorded over multiple days, the final biomarker for each participant was the mean value across all recorded days. To compute each biomarker for a given day, at least 80% of the 96 expected data points per 24-hour period were required. Only participants with at least two such days were included.

The hypoglycaemic AUC was computed as the area between 70 mg/dL and the glycaemic values during hypoglycaemic episodes. A hypoglycaemic episode is defined as any period in which glucose fell below 70 mg/dL for at least two consecutive measurements, both recorded within a 30-minute interval (Supplementary Figure S1). A larger AUC corresponds to a more severe hypoglycaemic episode; for example, given the same duration, an episode with lower glucose values produces a larger AUC, indicating greater severity.

### Data analysis

Data analysis was conducted using R 4.4.0 and Python 3.11.2. Statistical significance was set at *p* < 0.05. Continuous variables are presented as median ± standard deviation (SD). Correlation analyses were performed between glycaemic biomarkers and both illness duration and BMI, with Spearman correlation coefficients and corresponding *p*-values calculated for each analysis. Additionally, a linear regression analysis was applied to evaluate the effects of BMI, illness duration, and EDI-2 total score on the glycaemic biomarkers.

## Results

**Figure 2.**
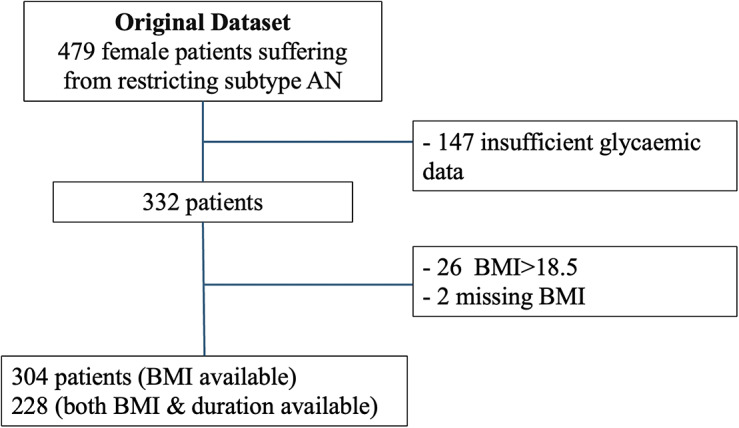
Flowchart. This study involved two separate analyses focusing on both illness duration and BMI. Patients were excluded if they had missing recordings or if less than 80% of the expected data points were available for at least one night. AN, anorexia nervosa.

Initially, 479 patients from the database were eligible (Figure 2). Of these, 304 were included in the BMI analysis and 228 in the illness duration analysis, while 147 were excluded due to missing glycaemic data. On average, patients were monitored for 4.8 ± 0.5 days, and 76 ± 13% of these days met the criteria for biomarker computation. The characteristics of the sample for each analysis (BMI and illness duration) are described in Table 1.

**Table 1.** Sample characteristics for BMI and illness duration analysis

	BMI analysis (*n* = 304)			Illness duration analysis (*n* = 228)			
Characteristics	Median		IQR	Median		IQR	*p*
Age (years)	24		12	24		12	0.92
BMI (kg/m^2^)	15.1		1.32	15.1		2.27	0.8
Illness duration (months)	60 (*n* = 228)		120	60		132	1
EDI–2	88 (*n* = 294)		58.5	90 (*n* = 225)		67	0.68

Abbreviations: BMI, body mass index; EDI-2, Eating Disorder Inventory; IQR, interquartile range.

### Effect of BMI on glycaemia

**Figure 3.**
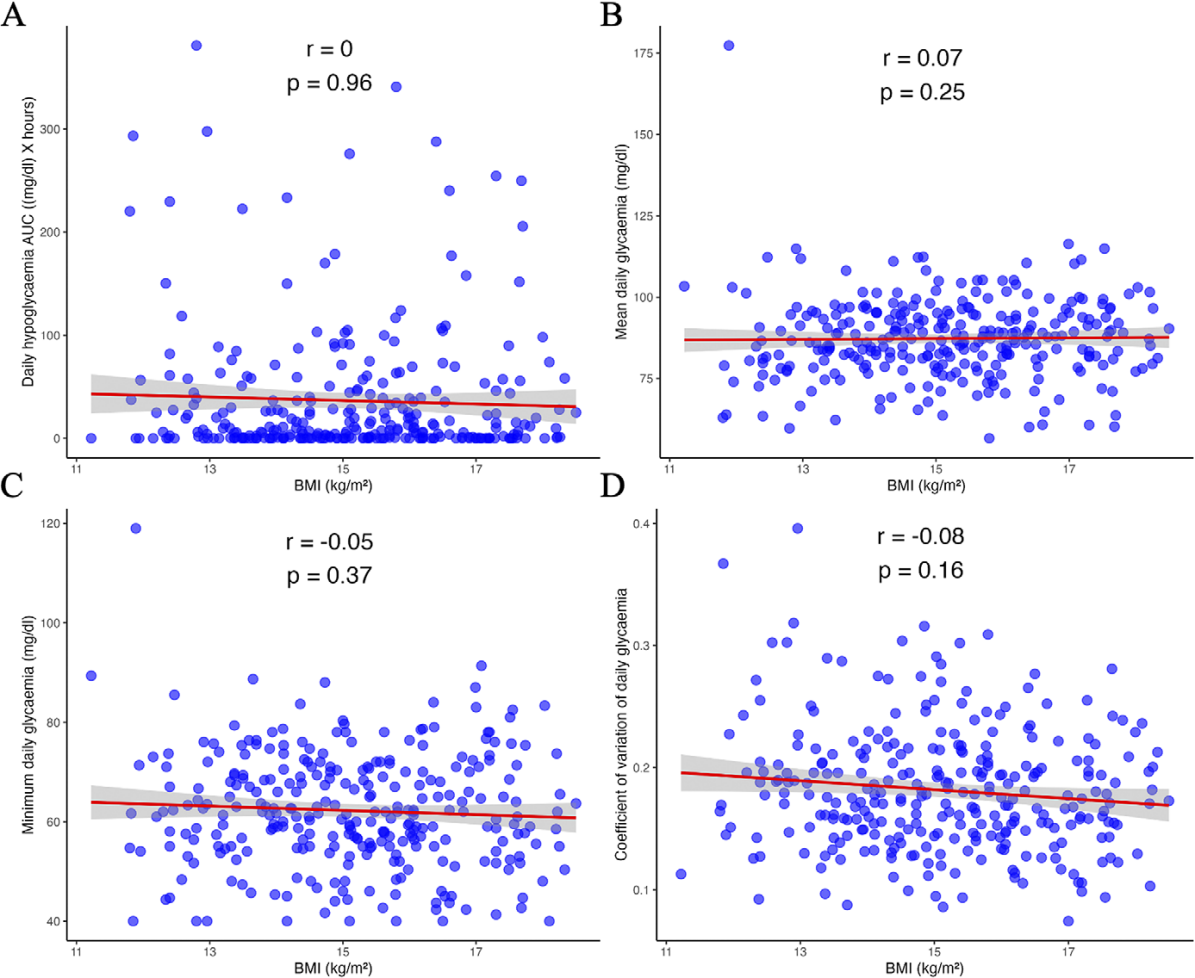
Correlation between BMI (body mass index) and glycaemic biomarkers: hypoglycaemic area under the curve (A), mean glycaemia (B), minimum glycaemia (C), and glycaemic coefficient of variation (D). AUC, area under the curve; *r*, Spearman’s rho.

No significant correlations were found between the glycaemic biomarkers and current BMI: hypoglycaemic AUC (*r* = 0.00, *p* = 0.96), mean glycaemia (*r* = 0.07, *p* = 0.25), minimum glycaemia (*r* = −0.05, *p* = 0.37) and glycaemic coefficient of variation (*r* = −0.08, *p* = 0.16; Figure 3). When BMI was considered as an indicator of severity according to DSM-5 criteria, no effect of BMI on glycaemic biomarkers was observed (Supplementary Table S1). Similar results were obtained for the analysis restricted to the nocturnal period (Supplementary Table S2).

### Effect of illness duration on glycaemia

**Figure 4.**
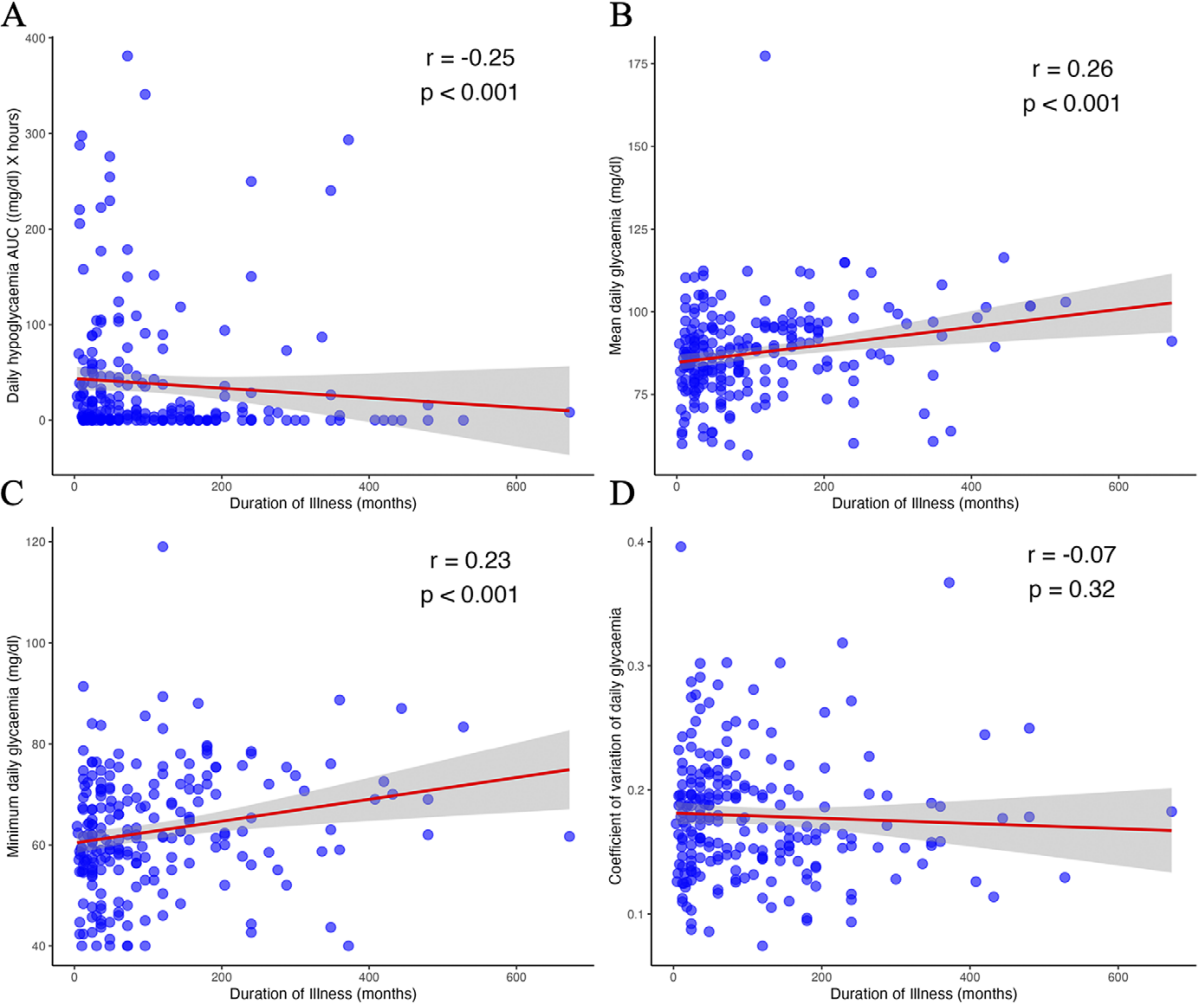
Correlation between illness duration and glycaemic biomarkers: hypoglycaemic area under the curve (A), mean glycaemia (B), minimum glycaemia (C), and glycaemic coefficient of variation (D). AUC, area under the curve; *r*, Spearman’s rho.

Significant positive correlations were observed between illness duration and both mean glycaemia (*r* = 0.26, *p* < 0.001) and minimum glycaemia (*r* = 0.23, *p* < 0.001; Figure 4). In contrast, hypoglycaemic AUC was negatively correlated with illness duration (*r* = −0.25, *p* < 0.001). No significant correlation was found for the glycaemic coefficient of variation (*r* = −0.07, *p* = 0.32). Similar results were obtained for the nocturnal period analysis (Supplementary Table S2).

### Effect of BMI, illness duration, and psychopathology severity on glycaemic indicators

A linear regression analysis was performed to assess the impact of BMI, illness duration, and clinical severity (EDI-2 score) on glycaemic indicators. For mean glycaemia, the overall model explained 5% of the variance (*R*^2^ = 0.07, adjusted *R*^2^ = 0.05, *p* < 0.01). Illness duration was a significant predictor (*β* = 0.027, *t* = 3.426, SE = 0.008, *p* < 0.001), whereas BMI (*β* = −0.417, *t* = −0.751, SE = 0.555, *p* = 0.45) and EDI-2 score (*β* = −0.032, *t* = −1.603, SE = 0.02, *p* = 0.11) were not.

For minimum glycaemia, the overall model explained 6% of the variance (*R*^2^ = 0.08, adjusted *R*^2^ = 0.06, *p* < 0.001). BMI remained a non-significant predictor (*β* = −0.737, *t* = −1.504; SE = 0.490, *p* = 0.134), while illness duration (*β* = 0.022, *t* = 3.201, SE = 0.007, *p* < 0.01) and EDI-2 score (*β* = −0.037, *t* = −2.074, SE = 0.18, *p* = 0.039) were significant predictors.

The overall models for hypoglycaemic AUC and glycaemic coefficient of variation were not significant (*p* = 0.051 and *p* = 0.75, respectively).

Overall, the models indicated that longer illness duration was associated with higher mean and minimum glycaemia, while higher EDI-2 total scores were associated with lower minimum glycaemia.

## Discussion

In a relatively large cohort of adult female patients with restricting subtype AN monitored using CGM, we observed that hypoglycaemia was significantly influenced by prolonged illness duration, marginally influenced by psychopathological severity, and not affected by current BMI.

To the best of our knowledge, this is the first study to apply CGM to a large group of patients with AN, opening a novel avenue for understanding glycaemic regulation in this population. Previous studies have more frequently used CGM to assess binge-eating and vomiting episodes or meal consumption in BN or BED [12–14]. Only two studies have investigated CGM measures in patients with AN. Germain et al. applied CGM sensors to 28 adult females with AN (19 restricting and 4 binge-purging subtype) during a five-day hospitalization, with daily calibration, to monitor glycaemic patterns [16]. They reported chronic mild hypoglycaemia in their sample, with 21% of the monitored time below 70 mg/dL. The authors supported the use of CGM in AN and defined night-time ranges. Similar observations were made by Uotani et al. in a sample of outpatients with AN (4 restricting, 9 binge-purging subtype), using the same CGM device; in this study, no hypoglycaemic episodes (<60 mg/dL) were observed in patients with the restricting subtype during the five-day monitoring period [15].

In our study, BMI did not appear to influence the occurrence of hypoglycaemia: patients with severe AN and low BMI did not experience more hypoglycaemic episodes than those with higher BMI. One potential explanation is a metabolic adaptation that limits severe hypoglycaemia [20]. Alternatively, the lack of association could reflect the absence of extremely underweight patients in our sample. Notably, our cohort comprised patients seeking care outside emergency settings and did not include any patients with a BMI below 12 kg/m^2^, as these individuals are typically referred to specialized nutrition services. However, hypoglycaemia is primarily observed in patients requiring acute care and is more likely to occur in those with severely impaired nutritional status, such as low prealbumin levels [21]. Consequently, hypoglycaemia remains uncommon in non-emergency hospitalized AN populations, with only 7% of patients with the restricting subtype affected in the largest cohort reported to date [22]. Therefore, the lack of patients at the extreme low end of BMI in our sample may have limited the likelihood of observing hypoglycaemic episodes and reduced the statistical power to detect a correlation with BMI.

Our results also show that illness duration strongly influences glycaemia. Germain et al. did not find an effect of illness duration, but their study was based on a smaller sample of inpatients with AN [16], who had a lower average BMI. Inpatient treatment involves increased monitoring of eating behaviour, which reduces variability and limits contrasts. In our study, prolonged illness was associated with a reduction in hypoglycaemic AUC and an increase in mean and minimum glycaemia. The positive correlations between illness duration and mean and minimum glycaemia may support our hypothesis of metabolic adaptation over the course of the illness. Additionally, the negative correlation between hypoglycaemic AUC and illness duration suggests a progressive reduction in hypoglycaemia as the illness advances.

Illness duration was a significant predictor of both mean and minimum glycaemia, showing a positive association with both biomarkers. By contrast, BMI did not significantly affect any glycaemic measures, while the EDI-2 score was a significant predictor of minimum glycaemia. These findings suggest that AN-related psychopathology, as measured by the EDI-2, is linked to glucose regulation. Further research is warranted to characterize the relationships between specific pathological dimensions, such as emotional regulation and asceticism, and glycaemic regulation.

Our preliminary findings echo recent discoveries highlighting the role of metabolic factors in the emergence and maintenance of AN [4, 20]. Indeed, the first large-scale genetic analyses have unexpectedly implicated glycaemic metabolism, through various parameters such as insulin resistance (HOMA-IR), fasting insulin, beta-cell function, and fasting glucose [5]. In particular, negative genetic correlations with insulin resistance-related conditions (−0.71 < *r* < −0.55) appear to be specific to AN and more pronounced than in other psychiatric disorders [23]. Furthermore, these negative genetic correlations with glucose and insulin metabolism remain significant after controlling for BMI [24] and in larger samples [3].

Beyond genetic associations, several metabolic adaptations to starvation may help maintain euglycaemia [20]. Elevated cortisol, inducing insulin resistance, increased growth hormone promoting gluconeogenesis, and elevated plasma ghrelin levels may all contribute to such glycaemic adaptations [20, 25, 26]. Preclinical models of AN support the coexistence of hyperghrelinemia with euglycaemia under chronic food deprivation. For example, ghrelin-deficient mice exhibit a drop in glycaemia [27]. Further studies integrating both clinical and preclinical approaches are needed to elucidate these complex regulatory mechanisms more precisely.

Several limitations should be acknowledged. First, our clinical data were recorded in a real-world setting from a relatively large sample, but information regarding daily food intake, physical activity, and other factors influencing glycaemia was not available. Focusing exclusively on the restricting subtype partially mitigates the impact of these behaviours. Second, the measurement of illness duration relies on an accurate assessment of the age at onset of AN, which can be challenging, even though the MINI facilitated this process. Third, CGM data were collected in a single clinical unit and concerned only adult females with restricting subtype AN, limiting the generalizability of our findings. The absence of adolescent patients in our sample restricts analysis of age-related effects, especially given that 50% of AN cases have an age at onset before 18 years [1]. Furthermore, as our sample consisted of outpatients, it did not include patients with extremely severe AN, which may partly explain the lack of correlation between BMI and hypoglycaemia discussed above. However, our protocol is not applicable to inpatients, as real-life CGM monitoring is not feasible in a hospital setting. Fourth, it was not possible to control for recent weight fluctuations, which could confound our results; patients with a more recent disorder may have experienced greater weight fluctuations in the studied period, potentially affecting glucose metabolism. Fifth, several technical limitations should be considered. The CGM devices were designed primarily to detect and prevent hyperglycaemia in diabetes and may be less accurate for assessing low glucose variations. Additionally, because the device takes time to calibrate, the first hours of recording were excluded from our analyses, while other studies adopt an even more conservative approach by excluding the first 24 hours [15]. Finally, the cross-sectional design may present limitations. Selecting patients with the restricting subtype does not preclude a later transition to the binge-purging subtype, which is part of the clinical course of AN in a significant proportion of patients [28]. Nonetheless, patients with long-lasting restricting subtype AN can be considered a specific and clinically relevant subgroup.

## Conclusion

The use of CGM in real-world settings across more than 300 patients with AN provided detailed, continuous glycaemic data over five-day periods. Our findings indicate that illness duration, rather than current BMI, is significantly associated with variations in glycaemic profiles in adults with AN, with psychopathological severity also exerting some influence. Given its ease of implementation in daily life, CGM may help identify novel glycaemic biomarkers linked to disease progression and psychopathology, and holds promise as a valuable tool for further exploring the complex metabolic mechanisms underlying AN. Although our results could be interpreted as suggesting potential metabolic adaptations over the course of the illness, this hypothesis requires further investigation. Larger, longitudinal studies using CGM are warranted to deepen our understanding of metabolic changes and their clinical significance in AN.

## Supporting information

10.1192/j.eurpsy.2025.10144.sm001 Pulini et al. supplementary material Pulini et al. supplementary material

## Supplementary material

The supplementary material for this article can be found at http://doi.org/10.1192/j.eurpsy.2025.10144.
