# Supplementary material for: Drivers of hypoglycaemia in anorexia nervosa: Clinical severity, BMI, and illness duration
Source: Eur Psychiatry. 2025 Dec 17;69(1):e8. doi: 10.1192/j.eurpsy.2025.10144 (PMC12816928; doi:10.1192/j.eurpsy.2025.10144)
Supplement: Pulini et al. supplementary material [file S0924933825101442sup001.docx]

## Supplementary material

**Method**

## Nocturnal glycaemic biomarkers

## To replicate the analysis by Germain et al, we examined nocturnal glucose variation between 23:00 and 05:00. As glucose levels were recorded every 15 minutes, up to 24 data points were available per night. To calculate each biomarker, at least 80% of the expected data points within this time window were required for each night.

## Analysis regarding BMI severity groups

BMI (kg/m²) effects were analysed using DSM-5 severity categories: mild (17–18), moderate (16–17), severe (15–16), and extreme (<15).

##
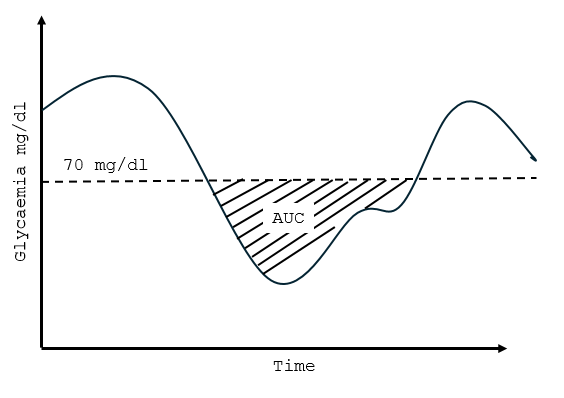


**Figure S1**. Method of AUC calculation for hypoglycaemic episodes.

**Table S1.** Sample characteristics depending on BMI severity

|  |  | BMI Severity | | | | | | | | | | | | | | |  |  |  |  |
| --- | --- | --- | --- | --- | --- | --- | --- | --- | --- | --- | --- | --- | --- | --- | --- | --- | --- | --- | --- | --- |
|  |  | Extreme (N=141) | | |  | Severe (N=66) | | |  | Moderate (N=48) | | |  | Mild (N=49) | | |  | p |  |  |
| **Characteristics** |  | Median |  | IQR |  | Median |  | IQR |  | Median |  | IQR |  | Median |  | IQR |  |  |  |  |
| Age (years) |  | 25 |  | 12 |  | 23 |  | 8.75 |  | 24.5 |  | 12.25 |  | 24 |  | 13 |  | 0.75 |  |  |
| BMI (kg/m²) |  | 13.9 |  | 1.32 |  | 15.43 |  | 0.52 |  | 16.37 |  | 0.44 |  | 17.55 |  | 0.44 |  | 0 |  |  |
| Illness duration (months) |  | 60 (n=106) |  | 120 |  | 48 (n=49) |  | 82 |  | 84 (n=42) |  | 153 |  | 84 (n=31) |  | 144 |  | 0.75 |  |  |
| EDI-2 |  | 77.5 (n=138) |  | 58.5 |  | 93 (n=63) |  | 55 |  | 92 |  | 76.75 |  | 109 (n=45) |  | 52 |  | 0.001 |  |  |
| **Glycaemic biomarkers** |  |  |  |  |  |  |  |  |  |  |  |  |  |  |  |  |  |  |  |  |
| Nocturnal hypoglycaemia AUC ((mg/dl) X hours) |  | 2.75 |  | 13.88 |  | 6.38 |  | 20.94 |  | 2.39 |  | 12.66 |  | 3.56 |  | 15.09 |  | 0.15 |  |  |
|  |  |  |  |  |  |  |  |  |  |  |  |  |  |  |  |  |  |  |  |  |
| Mean nocturnal glycaemia (mg/dl) |  | 82.61 |  | 18.45 |  | 79.35 |  | 14.92 |  | 83.67 |  | 15.01 |  | 82.55 |  | 16.24 |  | 0.22 |  |  |
|  |  |  |  |  |  |  |  |  |  |  |  |  |  |  |  |  |  |  |  |  |
| Minimal nocturnal glycaemia (mg/dl) |  | 68 |  | 14.25 |  | 63 |  | 13.5 |  | 67.13 |  | 13.56 |  | 66 |  | 22.25 |  | 0.1 |  |  |
|  |  |  |  |  |  |  |  |  |  |  |  |  |  |  |  |  |  |  |  |  |
| Nocturnal glycaemia coefficient of variation |  | 0.11 |  | 0.07 |  | 0.12 |  | 0.06 |  | 0.12 |  | 0.05 |  | 0.11 |  | 0.06 |  | 0.8 |  |  |
|  |  |  |  |  |  |  |  |  |  |  |  |  |  |  |  |  |  |  |  |  |
| Daily hypoglycaemia AUC ((mg/dl) X hours) |  | 6.29 (n=137) |  | 38.67 |  | 16.25 (n=64) |  | 44.63 |  | 6.13 (n=47) |  | 38.35 |  | 8.19 (n=48) |  | 44.06 |  |  |  |  |
|  |  |  |  |  |  |  |  |  |  |  |  |  |  |  |  |  |  |  |  |  |
| Mean daily glycaemia (mg/dl) |  | 86.09 (n=137) |  | 15.16 |  | 86.40 (n=64) |  | 15.02 |  | 87.96 (n=47) |  | 11.15 |  | 89.39 (n=48) |  | 15.45 |  |  |  |  |
|  |  |  |  |  |  |  |  |  |  |  |  |  |  |  |  |  |  |  |  |  |
| Minimal daily glycaemia (mg/dl) |  | 62.67 (n=137) |  | 16 |  | 58.67 (n=64) |  | 12.75 |  | 62 (n=47) |  | 13.50 |  | 60.75 (n=48) |  | 19.67 |  |  |  |  |
|  |  |  |  |  |  |  |  |  |  |  |  |  |  |  |  |  |  |  |  |  |
| Daily glycaemia coefficient of variation |  | 0.18 (n=137) |  | 0.06 |  | 0.19 (n=64) |  | 0.06 |  | 0.17 (n=47) |  | 0.06 |  | 0.17 (n=48) |  | 0.06 |  |  |  |  |
| BMI severity: mild when BMI ϵ [17:18.5[. moderate when BMI ϵ [16:17[. severe when BMI ϵ [15:16[. extreme when BMI<15 Reported values correspond to Median (IQR). BMI: body mass index; EDI: eating disorder inventory; IQR: interquartile range; p: p-value. | | | | | | | | | | | | | | | | | | | |  |
|  |  |  |  |  |  |  |  |  |  |  |  |  |  |  |  |  |  |  |  |  |

|  | **Glycaemic biomarkers (00:00-23:59)** | | | |
| --- | --- | --- | --- | --- |
|  | hypoglycaemic AUC | mean | minimum | coefficient of variation |
| **BMI** | r = 0.00, p = 0.96 | r = 0.07, p = 0.25 | r = -0.05, p = 0.37 | r = -0.08, p = 0.16 |
| **Illness Duration** | r = -0.25, p < 0.001 | r = 0.26, p < 0.001 | r = 0.23, p < 0.001 | r = -0.07, p = 0.32 |
|  | **Nocturnal glycaemic biomarkers (23:00-05:00)** | | | |
| **BMI** | r = 0.01, p = 0.89 | r = 0.01, p = 0.91 | r = -0.04, p = 0.50 | r = 0.00, p = 0.99 |
| **Illness Duration** | r = -0.20, p < 0.01 | r = 0.29, p < 0.01 | r = 0.24, p < 0.01 | r = 0.12, p = 0.08 |

**Table S2.** Nocturnal compared to 24-hour glycaemic biomarkers. *AUC: area under the curve, r: Spearman’s rho*
